# Effect of 12 weeks of detraining and retraining on the cardiorespiratory fitness in a competitive master athlete: a case study

**DOI:** 10.3389/fphys.2024.1508642

**Published:** 2024-11-22

**Authors:** Romuald Lepers, Adrien Mater, Hervé Assadi, Nadège Zanou, Vincent Gremeaux, Nicolas Place

**Affiliations:** ^1^ Inserm U1093, CAPS Université de Bourgogne, Faculté des Sciences du Sport, Dijon, France; ^2^ Institute of Sport Sciences, Faculty of Biology and Medicine, University of Lausanne, Lausanne, Switzerland; ^3^ Department of Biomedical Sciences, Faculty of Biology and Medicine, University of Lausanne, Lausanne, Switzerland; ^4^ Department of Sports Medicine, Swiss Olympic Medical Center, Lausanne University Hospital, Lausanne, Switzerland

**Keywords:** endurance, triathlon, running, cycling, reconditioning, VO_2max_, peak power output, running economy

## Abstract

**Purpose:**

This study examined the physiological effects of 12 weeks of detraining and retraining in a highly trained master triathlete (age 53.8 years).

**Methods:**

Variables associated with swimming, cycling, and running performance, including $\dot{V}$ O_2max_, peak power output (PPO), gross cycling efficiency (CE), running maximal aerobic velocity (MAV), running economy (RE), muscle strength, and body composition were assessed before the last race of the season (baseline), after 12 weeks of detraining, and after 12 weeks of retraining.

**Results:**

Detraining resulted in a 9.1% and 10.9% decrease in relative $\dot{V}$ O_2max_ for cycling and running, respectively. PPO and MAV declined by 12.7% and 8.6%, respectively. After detraining, CE decreased by 6.2%, and RE was 22% higher than the baseline. The maximal strength capacity of the knee extensor muscles decreased by an average of 8.2%. Body fat percentage increased from 10.5% to 13.8%, while lean mass decreased by 2.2 kg. After retraining, almost all variables returned to baseline or even slightly increased, except RE and lean mass, which did not return to baseline.

**Conclusion:**

After 12 weeks of detraining, a lifelong master triathlete can regain his cardiorespiratory fitness (i.e., $\dot{V}$ O_2max_) with 12 weeks of progressive and structured retraining, but his running economy and lean mass remain slightly depressed.

## Introduction

The time needed to return to a fitness level close to the initial state after a prolonged training cessation (e.g., more than a month) is an important issue for many athletes. Detraining is characterized by marked alterations in the cardiorespiratory system (e.g., reduction in maximal oxygen uptake - $\dot{V}$ O_2_max) and metabolic patterns during exercise, and depends on several factors, such as cessation duration, previous training load and initial level of the athletes (Mujika and Padilla, 2000; Barbieri et al., 2024).

Aging is associated with progressive reductions in cardiovascular and neuromuscular function, contributing to the decline in the physiological determinants of endurance performance with advancing age. For example, maximum cardiorespiratory fitness, measured by maximal oxygen uptake, decreases by 0.5%–1% per year starting at age 30, primarily due to lower cardiac output resulting from a reduction in maximum heart rate (0.7 beats per minute per year) and a decrease in muscle mass (Trappe et al., 1996). Running economy at a fixed running speed has also been reported to decrease with age in some studies (e.g., dos Anjos Souza et al., 2023), though not all (e.g., Quinn et al., 2011), with this decline partially attributed to reductions in tendon stiffness (Karamanidis and Arampatzis, 2006), which, in turn, decreases the mechanical efficiency of the muscle (Bohm et al., 2019). Finally, the metabolic rate at which the lactate threshold occurs has been reported to decrease (Wiswell et al., 2000) or remain unchanged with age (Allen et al., 1985). While master athletes experience notable declines in cardiovascular function and endurance capacity after periods of detraining, they may maintain certain physiological adaptations acquired through lifelong training, potentially mitigating the effects of detraining (Barbieri et al., 2024).

Endurance exercise training induces, among other adaptations, increases in stroke volume and muscle oxidative capacity, resulting in an increase in $\dot{V}$ O_2_max. There is little data in the literature on how rapidly these parameters adapt when highly trained individuals, who have exercised regularly for years, stop training for prolonged periods. The review by Burtscher et al. (2022) based on fewer than ten studies, suggests an almost linear decrease in $\dot{V}$ O_2max_ of up to about 20% following training cessation periods ranging from a few days to 12 weeks. For example, Coyle et al. (1984) showed that in young (≈30 years old) highly endurance-trained athletes, the reduction in absolute $\dot{V}$ O_2max_ could reach 16% after 12 weeks of training cessation. Although master athletes (>40 years old) have increasingly participated in endurance events in recent decades (Lepers and Stapley, 2016), we still lack comprehensive knowledge about the process of their detraining and retraining. Nichols et al. (2020) observed that following a 4-week detraining period (due to a collarbone fracture), the $\dot{V}$ O_2max_ of a female master (49.5 years old) cyclist decreased by 26%. Interestingly, after just 6 weeks of retraining, her $\dot{V}$ O_2max_ returned to its preinjury value, while peak power output (PPO) was still 6% lower, and it took 11 weeks to reach the preinjury value.

Conducting studies to examine the effects of detraining and retraining in competitive, well-trained athletes is difficult, as these athletes are often reluctant to voluntarily stop their training for a long period (e.g., >4 weeks). Consequently, even case reports represent valuable information on changes in physical fitness after periods of detraining and retraining.

In a previous case report, we demonstrated that a 12-week detraining program could benefit a well-trained master athlete by enhancing their muscle metabolic phenotype and aerobic capacity following a retraining protocol (Zanou et al., 2024). At the muscle level, detraining was associated with reduced levels of mitochondrial biogenesis and oxidative phosphorylation (OXPHOS) proteins. Conversely, it led to an increase in the levels of the ryanodine receptor type 1 (RyR1) protein, myosin heavy chain fast-twitch (fast MHC), and Sarco/Endoplasmic reticulum Ca2+ ATPase type 1 (SERCA) protein. In contrast, retraining elevated not only mitochondrial proteins but also glycolytic proteins beyond baseline levels, likely improving skeletal muscle metabolism. In this companion article, we describe the effects of a 12-week detraining period followed by a retraining period on cardiorespiratory fitness, muscular strength, and body composition in the same highly trained male master triathlete.

## Methods

### Subject

At the first evaluation date, the subject was a 53.8-year-old French Caucasian. He had been racing triathlons since the age of 20. At the peak of his amateur career, he twice finished in the top 150 at the Hawaii Ironman triathlon championship. Since entering the master category (>40 years old), he has regularly placed in the top 5 of his categories in Ironman 70.3 events. For the past 20 years, he has maintained a regular training program, with no break of more than 2 weeks in a row each year. His annual training volume has remained relatively constant at around 500 h per year (120 h swimming, 270 h cycling, and 110 h running). In the summer of 2022, he voluntarily decided to take a 3-month break after his last scheduled competition on 5 September 2022 (ÖTILLÖ, Swimrun World Championship in Sweden, consisting of a 10 km open-water swim and 65 km trail run, where he placed 42nd in men’s team category in 10 h 01 min). In the 3 months leading up to his last competition, he trained an average of 10.8 h per week, consisting of 32% swimming, 38% cycling, and 30% running.

### Design

Baseline data were collected in the 2 weeks leading up to the cessation of training. The day after his last competition, the athlete stopped all structured training and only engaged in minimal physical activity for 12 weeks. He walked twice a week for 30 min at a slow pace and completed 15 min of core training twice a week (e.g., forearm plank, side bend, hip dip). Re-testing was carried out during the week following the 12-week detraining period. The athlete then began a structured retraining program for 12 weeks, gradually progressing from approximately 50% of his usual training volume to 100% over the following 6 weeks. By the seventh week of retraining, he had reached his previous regular training regimen (10–12 h per week). During the retraining period, his training volume was divided as follows: 28% swimming, 47% cycling, and 25% running. The athlete showed no abnormal symptoms or injuries when he resumed training and did not change his diet.

### Measurements

The master triathlete carried out swimming, cycling, and running tests.

### Swimming test

The participant performed a 400 m freestyle test in a 50 m length pool.

### Cycling test

The participant performed an incremental exercise test on an electrically braked cycle ergometer (Lode Excalibur, Groningen, Netherlands) equipped with automatic pedals. This test was used to determine gross cycling efficiency (CE), $\dot{V}$ O_2max_, maximal ventilation ( $\dot{V}$ E_max_), maximal heart rate (HR_max_), and peak power output (PPO). The test began at 100 W for 2 min, with the power output was increased by 25 W every 2 min until volitional exhaustion, except at the 200 W stage, which lasted 5 min in order to assess cycling efficiency. PPO was defined as the highest power completed for 2 min. If the athlete stopped before the end of the 2-min stage, PPO was calculated proportionally to the time spent in the last stage. CE was calculated at 200 W using the following formula: *CE*= (*PO* × Δ*t*). ( $\dot{V}$ *O*_2_ × *O*_2_ *equiv*.) ^−1^ where PO is the power output (W), Δt is the duration of the stage (s), $\dot{V}$ O_2_ is the oxygen consumption (L.min^−1^) and O_2_ equiv. is the energetic equivalent associated with respiratory ratio (kJ.L^−1^) (Coyle et al., 1992).

### Running test

The subject performed a continuous incremental exercise on a Pulsar 3p® treadmill (h/p/cosmos sports and medical gmbh, Traunstein, Germany). This test allowed us to determine running economy (RE), VO_2max_, $\dot{V}$ E_max_, HR_max_, and maximal aerobic velocity (MAV). The initial velocity was set at 8 km h^−1^ and increased by 0.5 km h^−1^ per minute until the subject could no longer maintain the speed. An exception was made at the 12 km h^−1^ stage, which lasted 5 min to assess running economy. The highest running velocity reached during the test was considered the MAV. RE was calculated at 12 km h^−1^ using the formula: RE = $\dot{V}$ O_2_.V^−1^ where $\dot{V}$ O_2_ is the oxygen consumption (mL.min^−1^.kg^−1^) and V is the running velocity (km.min^−1^) (di Prampero, 1986).

For the calculation of CE and RE, the power output and running velocity were based on the levels the athlete could maintain throughout the 180 km cycling and 42 km running sections of an Ironman triathlon. During the stages where CE and RE were calculated, the RER values were below 1.0, ranging between 0.81 and 0.94.

### Cardiorespiratory responses

Heart rate and $\dot{V}$ O_2_ were measured using a chest strap heart rate monitor (model V800; Polar Electro Oy, Kempele, Finland) and a metabolic analyzer K5 (COSMED, Rome, Italy), respectively. The gas analyzer was calibrated and used according to the company guidelines in a controlled environment with constant temperature and humidity. The maximal effort was confirmed based on the following criteria: a plateau in the $\dot{V}$ O_2_–velocity/power relationship with $\dot{V}$ O_2_ increasing by less than 2 mL kg^−1^.min^−1^ despite increases in velocity or power output, a peak respiratory exchange ratio (RER) greater than 1.10, or peak heart rate within 10 beats per minute of the age-predicted maximum. $\dot{V}$ O_2max_ was defined as the highest value sustained for 30 s.

### Knee extensor muscle strength

The participant performed maximal isometric and isokinetic voluntary knee extensor contractions with his right leg in concentric mode at 60°.s^−1^, 120°.s^−1^, and 180°.s^−1^. He was seated on an isokinetic dynamometer (System pro 4, Biodex Medical System, New York) with a 90° hip flexion and the knee joint axis in line with the rotation axis of the dynamometer. At least two maximal voluntary contractions of each contraction mode and angular velocity were performed (more if the difference between the peak torque of two consecutive contractions was greater than 5%). The trial with the greatest peak torque was analyzed. The participant also performed a Thorstensson Test (Thorstensson and Karlsson, 1976) to assess muscular endurance. Briefly, 50 maximal isokinetic voluntary knee extensor contractions were repeated at 180°.s^−1^, and a fatigue index over 50 contractions was calculated as: (last 5-first 5)/first 5 × 100. The participant was instructed to give an all-out effort during each concentric action and to relax his leg during flexion back to the starting position.

Finally, three squat jumps (SJ) and three countermovement jumps (CMJ), each separated by 1-min recovery, were performed. The trial with the greatest height was analyzed. The participant had to keep his hands on his hips, starting from a static standing position, leg straight for CMJ and leg fold at 90° for SJ. The subject was instructed to jump as high as possible. Jump performance was assessed using the My Jump app on an iPhone (Gençoğlu et al., 2023).

### Body composition

Body composition was measured via dual-energy x-ray absorptiometry (Lunar iDXA ME + 210430). There were no specific instructions regarding hydration and diet prior to the DXA or performance tests, but participants were asked to maintain the same dietary routine before each testing session.

## Results

**FIGURE 1:**
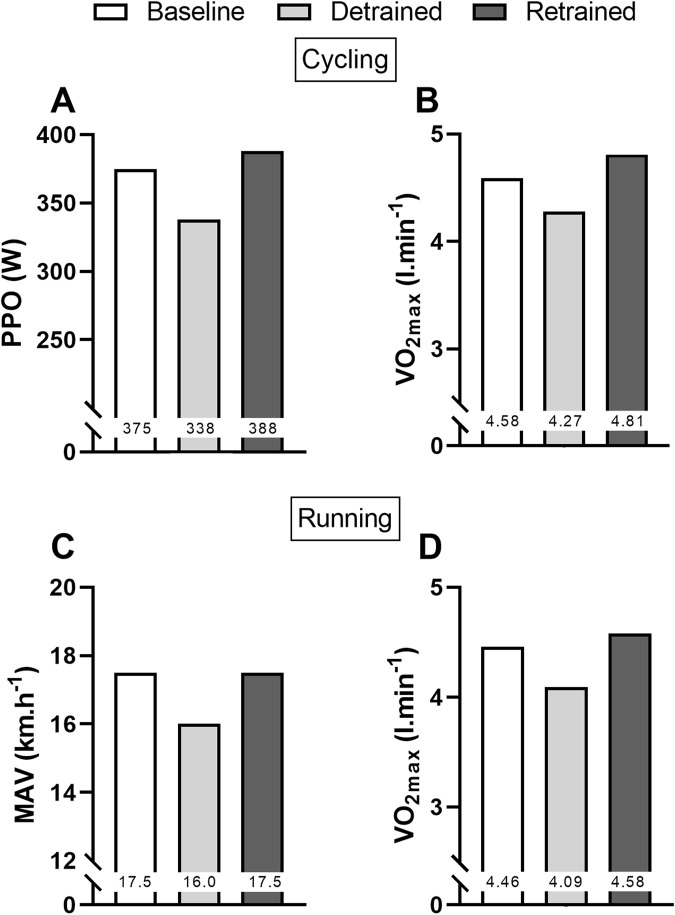
Values of peak power output (PPO) **(A)** and absolute maximal oxygen uptake (VO_2max_) **(B)** for cycling, and maximal aerobic velocity (MAV) **(C)** and absolute VO_2max_ **(D)** for running at baseline, after 12 weeks of training cessation (detrained), and after 12 weeks of retraining (retrained).

Figure 1 shows that absolute $\dot{V}$ O_2max_ decreased by 6.8% for the cycling test and 8.5% for running test after the 12-week detraining period, while PPO for cycling and MAV for running decreased by 9.9% and 8.6%, respectively. When expressed relative to body mass, the reduction in $\dot{V}$ O_2max_ reached 9.1% for cycling and 10.9% for running, respectively (see Table 1). After 3 months of retraining, these variables improved considerably and were even slightly higher than baseline values. Indeed, compared to baseline values, absolute $\dot{V}$ O_2max_ was 2.4% higher for running and 5% higher for cycling, respectively. PPO increased by 3.5% after retraining, while MAV was similar to baseline. HR_max_ increased by 7-8 bpm after detraining and was slightly lower than baseline values after retraining (Table 1). $\dot{V}$ E_max_ decreased after detraining, particularly for running, and almost returned to baseline values after retraining.

**TABLE 1:** Swimming performance, physiological variables during the cycling and running incremental tests, and knee extensor muscle strength and jump performances at Baseline, after 3 months of detraining (Detrained), and after 3 months of retraining (Retrained). Body mass of the participant at the testing time: Baseline, 74.0 kg; Detrained, 76.1 kg; Retrained, 73.1 kg.

	Baseline T0	Detrained T1	Retrained T2	Diff. (%) T1-T0	Diff. (%) T2-T0
400-m Swimming test					
Time (min:s)	5:52	6:17	6:01		
Speed (m.s^−1^)	1.14	1.06	1.11	−6.7	−2.6
Cycling test					
$\dot{V}$ O_2_max (ml.min^−1^.kg^−1^)	62.0	56.2	65.8	−9.3	6.1
PPO (W.kg^−1^)	5.07	4.44	5.31	−12.7	4.7
HRmax (bpm)	175	183	171	4.6	−2.3
O_2_ pulse max (ml.kg^−1^.beat^−1^)	0.34	0.31	0.38	−8.8	11.8
$\dot{V}$ Emax (l.min^−1^)	159	154	156	−3.1	−1.9
RERmax	1.11	1.12	1.07	0.9	−3.6
CE at 200 W (%)	22.7	21.3	22.1	−6.2	−2.6
HR at 200 W (bpm)	117	140	111	19.7	−5.1
VE at 200 W (l.min^−1^)	61	65	62	6.6	1.6
Running test					
$\dot{V}$ O_2_max (ml.min^−1^.kg^−1^)	60.3	53.7	62.6	−10.9	3.8
HRmax (bpm)	172	179	171	4.1	−0.6
O_2_ pulse max (ml.kg^−1^.beat^−1^)	0.35	0.30	0.37	−14.3	5.7
$\dot{V}$ Emax (l.min^−1^)	140	124	138	−11.4	−1.4
RERmax	1.12	0.97	1.15	−13.4	2.7
RE at 12 km h^−1^ (ml.min^−1^.km^−1^)	186	227	219	22	17.7
HR at 12 km h^−1^ (bpm)	129	153	133	18.6	3.1
VE at 12 km h^−1^ (l.min^−1^)	64	88	76	37.5	18.7
Knee ext. strength and jumps					
MVC_iso_ (N.m)	182	175	177	−3.8	−2.7
MVC_60_ (N.m)	187	182	188	−2.7	0.5
MVC_120_ (N.m)	181	154	165	−14.9	−8.8
MVC_180_ (N.m)	169	150	166	−11.2	−1.8
Fatigue Index 180°.s^−1^ (%)	−26	−35	−28	34.6	7.7
SJ (cm)	22.9	19.9	22.5	−13.1	−1.7
CMJ (cm)	27.1	25.7	25.6	−5.2	−5.5

PPO: peak power output; $\dot{V}$ O_2_max: maximal oxygen uptake; HRmax: maximal heart rate; O_2_ pulse max: maximal O_2_ pulse defined as $\dot{V}$ O_2_max.HRmax^−1^. $\dot{V}$ Emax: maximal minute ventilation; RERmax: maximal respiratory exchange ratio; CE: Gross cycling efficiency. MAV: maximal aerobic velocity, RE: running economy. MVC_iso_, MVC_60_, MVC_120_, MVC_180_: knee extensor maximal voluntary contraction torque in isometric condition at 90° knee angle, and at 60°.s^−1^, 120°.s^−1^ and 180°.s^−1^, respectively. Fatigue Index 180°.s^−1^: fatigue index over 50 contractions at 180°.s^−1^ calculated as: (last 5-first 5)/first 5 × 100; SJ: squat jump; CMJ: counter movement jump.

Analysis at submaximal exercise intensities revealed that after 12 weeks of detraining, CE (at an intensity corresponding to 53% of baseline PPO) decreased by 6.2% and remained 2.6% below baseline after retraining (Table 1). Similarly, running economy (at an intensity corresponding to 68% of baseline MAV) was 22% higher after detraining and remained 17% higher after retraining compared to baseline. Heart rate and ventilation at these submaximal intensities were higher after detraining than baseline values for cycling and running. After retraining, these variables returned to baseline values for cycling but not for running.

Table 1 showed that, after detraining, maximal strength capacity of the knee extensor muscles decreased by an average of 8.2%, ranging from 2.7% to 14.9%, depending on the contraction mode. After retraining, maximal strength capacity remained slightly below baseline values (−3.2% on average). The fatigue index over 50 repeated dynamic contractions increased by 34.6% after detraining and remained higher by 7.7% after retraining compared to baseline values. After detraining, jumping performances decreased by an average of 9.2% (−5.2% for the CMJ and −13.1% for the SJ), respectively. After retraining, jumping performances remained slightly below baseline values (−3.6% on average).

Body fat percentage increased from 10.1% to 13.3% during detraining (Table 2). The participant gained 2.5 kg of fat and lost 2.2 kg of fat-free mass. The loss of fat-free mass was more pronounced in the trunk (−5.7%) than in the arms (−1%) and legs (−1.5%). After 12 weeks of retraining, the participant was lighter than at baseline, and his percentage of body fat had dropped to 8.7%. His body fat mass was 1.6 kg lower than the baseline value, and his free-fat mass was 1 kg lower. Bone mineral content remained unchanged during the detraining and retraining periods (Table 2).

**TABLE 2:** Body composition calculated by DEXA at Baseline, after 12 weeks of detraining (Detrained) and after 12 weeks of retraining (Retrained).

	Bodymass (kg)	Body fat (%)	Fat mass total (kg)	Fat-free mass total (kg)	Fat mass arms (kg)	Fat-free mass arms (kg)	Fat mass legs (kg)	Fat-free mass legs (kg)	Fat mass trunk (kg)	Fat-free mass trunk (kg)	Bone mineral content (kg)
Baseline	77.7	10.1	7.9	66.9	0.9	8.0	3.1	23.2	3.0	32.2	2.89
Detrained	78.1	13.3	10.4	64.8	1.1	7.9	3.6	22.9	4.7	30.4	2.89
Retrained	75.1	8.4	6.3	65.9	0.8	8.0	2.5	22.6	2.1	31.7	2.89

## Discussion

This study reports on changes in cardiorespiratory fitness, body composition and knee extensor maximal strength capacity of a highly trained 54-year-old master triathlete following 12 weeks of detraining and retraining. At baseline, his $\dot{V}$ O_2max_ was 62 mL kg^−1^.min^−1^, placing him above the 95th percentile of the age-related reference values provided by the ACSM’s Guidelines for Exercise Testing and Prescription (2021), indicating his high level of fitness.

The decrease in $\dot{V}$ O_2max_ (9%–10%) after 12 weeks of detraining was quite similar when measured in cycling or running and is in line with the lowest values reported earlier in literature for a similar detraining period (Burtscher et al., 2022; Coyle et al., 1984). Because no measurement was made during the 3 months of detraining, the kinetics of the decline in $\dot{V}$ O_2max_ could not be assessed. The decline in maximal O_2_ pulse - defined as $\dot{V}$ O_2_max.HRmax^−1^ by 8.8% for cycling and 14.3% for running suggests that maximal stroke volume and/or arteriovenous O_2_ difference decreased (Hagberg et al., 1985). Previous studies have suggested that a rapid decline in VO_2max_ occurs during the first 2–3 weeks with a less marked subsequent decline (Coyle et al., 1984; Klausen et al., 1981), but this is not always the case (Henriksson and Reitman, 1977). The initial phase of decrease in $\dot{V}$ O_2max_ in the first 2–3 weeks is thought to be due to a decrease in stroke volume, while the subsequent decrease would be attributed to a decrease in maximal arterio-venous oxygen difference (Coyle et al., 1984), as supported by the companion paper showing a decrease in mitochondrial biogenesis and oxidative phosphorylation proteins (Zanou et al., 2024).

When the participant was 40, his $\dot{V}$ O_2max_, PPO, and MAV were 71 mL kg^−1^.min^−1^, 425 W, and 20 km h^−1^, respectively. At the age of 53.8 (i.e., at baseline), these values were reduced by 12%–13%, corresponding to a loss of 7%–8% per decade, which is in line with existing literature, (around 7% per decade up to the age of 60 (Lepers et al., 2020; Valenzuela et al., 2020). Therefore, the 3 months of detraining (i.e., ∼10% loss of $\dot{V}$ O_2max_), was equivalent to aging by around 15 years, from a cardiorespiratory point of view.

After detraining, RE assessed at 12 km h^−1^ was much more altered than $\dot{V}$ O_2max_ (22% vs. 10.9%), and it was still 17.7% higher after retraining compared to baseline, while $\dot{V}$ O_2max_ returned to its initial value. This observation suggests that following a detraining period, the return to initial values is much slower for RE than for $\dot{V}$ O_2max_ (Jones, 2016). However, this needs to be confirmed with a larger sample of participants. After retraining, maximal leg strength capacity remained slightly depressed (−3%), which may partly explain the changes in RE. Other factors, such as biomechanical factors, could also play a role. For example, previous studies have shown that vertical, leg and knee stiffness were negatively correlated with RE (Li et al., 2021; Liu et al., 2022). However, older runners tend to show a less favorable balance between stiffness and reactive strength, possibly affecting performance and increasing energy costs at submaximal speeds (Jaén-Carrilo et al., 2021). It is worth noting that the proportion of training time devoted to running during the retraining period was lower compared to the pre-detraining period (25% vs. 30%), and running speeds were lower, especially at the beginning of the retraining period. The lower volume of running training may contribute to the reduced running economy seen after 12 weeks of retraining. Conversely, cycling efficiency was less affected by detraining than running economy (6.2% vs. 22%). The smaller reduction in energy cost for cycling compared to running during detraining can be attributed to the distinct characteristics of each activity. Running involves stretch-shortening cycles with eccentric muscle actions, whereas cycling is a non-weight-bearing activity that primarily uses concentric muscle actions and reduced osteoarticular stress. This observation suggests that after a detraining period, it takes longer for a triathlete to regain optimal energy cost in running compared to cycling, although this hypothesis requires further validation. Additionally, the relative time spent on cycling during the retraining phase was greater than during the pre-detraining period (47% vs. 38%). This increased focus on cycling during retraining may explain why cycling efficiency was nearly restored to baseline levels.

After detraining, the decline in maximum strength capacity was more pronounced for the concentric contractions at high contraction speeds (11%–15% at 120°.s^−1^ and 180°.s^−1^) compared with those at lower speeds (60°.s^−1^) or under isometric conditions (3%–4%). The ability to maintain a level of muscle torque over 50 dynamic contractions (estimated by the fatigue index) was much more diminished (35%) than the maximum strength capacity, suggesting that the subject was more fatigable after detraining. These results are consistent with the increase in fast MHC observed in the participant.^6^ The decline in strength capacity could be explained by the reduction in leg fat-free mass, which was reduced by 1.5% during the detraining period. The leg fat-free mass did not return to baseline after retraining, nor did strength capabilities completely return to baseline. It should be noted that total mass and body fat percentage were lower after retraining than at baseline, which contributed to the increased relative $\dot{V}$ O_2max_.

One of the limitations of this study is that the lactate threshold was not assessed. Lactate threshold has been identified as one of the main determinants of endurance, in addition to $\dot{V}$ O_2max_ and efficiency (i.e., the oxygen cost to generate a given running speed or cycling power output), (Joyner and Coyle, 2008). It would also have been interesting to monitor the time course of detraining and retraining, as well as other variables such as diet and physical activity, using accelerometry. Moreover, parameters such as voluntary activation level and muscle architecture would provide valuable insights into neuromuscular adaptations. Except the 400-m swim test, the athlete did not perform any cycling or running performance tests, such as a time to exhaustion at a given intensity or a time trial. During the triathlon racing season, starting 6 months after the end of the detraining period, the athlete’s performance level was in line with his usual level. Further studies are needed to better understand physiological changes after controlled periods of detraining and retraining in elite athletes with a high training volume (>20 h per week). Finally, the amplitude of changes between baseline and detraining or retraining for the physiological parameters, such as $\dot{V}$ O_2max_, reported in this case report, should be considered with caution, considering the reliability that is inherent to the measurement. Indeed, Hopkins et al. (2001) showed that the coefficient of variation for $\dot{V}$ O_2max_ and PPO could range from 1.5% to 5%.

### Practical applications

The results of this study suggest that after a detraining period of up to 12 weeks, well-trained endurance master athletes can regain their cardiorespiratory fitness (i.e., $\dot{V}$ O_2max_) in a period equivalent to the duration of the detraining. However, running economy seems to take longer to return to baseline levels. Additionally, because detraining leads to a significant decrease in muscle mass, master athletes should pay particular attention to regaining muscle mass and strength through specific training and diet during the retraining period.

## Conclusion

This case report illustrates the changes in cardiorespiratory fitness following 12 weeks of detraining and retraining in a lifelong endurance athlete following his competitive season. After a retraining period of the same duration as the detraining one, the master athlete reached and even exceeded his baseline level of cardiorespiratory fitness, but his running economy and lean mass remained slightly depressed.

## Data Availability

The raw data supporting the conclusions of this article will be made available by the authors, without undue reservation.
